# Microarray Profiling of Vaccination-Induced Antibody Responses to SARS-CoV-2 Variants of Interest and Concern

**DOI:** 10.3390/ijms232113220

**Published:** 2022-10-30

**Authors:** Julia Svetlova, Dmitry Gustin, Valentin Manuvera, Dmitriy Shirokov, Varvara Shokina, Kirill Prusakov, Konstantin Aldarov, Daria Kharlampieva, Daria Matyushkina, Julia Bespyatykh, Anna Varizhuk, Vassili Lazarev, Tatiana Vedekhina

**Affiliations:** 1 Federal Research and Clinical Center of Physical-Chemical Medicine, Malaya Pirogovskaya, 1a, 119435 Moscow, Russia; 2 Lomonosov Institute of Fine Chemical Technologies, MIREA—Russian Technological University, Vernadsky Avenue, 86, 119454 Moscow, Russia; 3 Moscow Institute of Physics and Technology (State University), 9, Institutskiy per., Moscow Region, 141701 Dolgoprudny, Russia; 4 K. I. Skryabin Moscow State Academy of Veterinary Medicine and Biotechnology, Akademika Skryabina Street, 23, 109472 Moscow, Russia; 5 Scientific Research Institute for Systems Biology and Medicine, Scientific Driveway, 18, 117246 Moscow, Russia

**Keywords:** microarrays, coronavirus infection, COVID-19, SARS-CoV-2, antibodies, Sputnik V, CoviVac

## Abstract

Mutations in surface proteins enable emerging variants of severe acute respiratory syndrome coronavirus 2 (SARS-CoV-2) to escape a substantial fraction of neutralizing antibodies and may thus weaken vaccine-driven immunity. To compare available vaccines and justify revaccination, rapid evaluation of antibody (Ab) responses to currently circulating SARS-CoV-2 variants of interest (VOI) and concern (VOC) is needed. Here, we developed a multiplex protein microarray-based system for rapid profiling of anti-SARS-CoV-2 Ab levels in human sera. The microarray system was validated using sera samples from SARS-CoV-2-free donors and those diagnosed with COVID-19 based on PCR and enzyme immunoassays. Microarray-based profiling of vaccinated donors revealed a substantial difference in anti-VOC Ab levels elicited by the replication-deficient adenovirus vector-base (Sputnik V) and whole-virion (CoviVac Russia COVID-19) vaccines. Whole-virion vaccine-induced Abs showed minor but statistically significant cross-reactivity with the human blood coagulation factor 1 (fibrinogen) and thrombin. However, their effects on blood clotting were negligible, according to thrombin time tests, providing evidence against the concept of pronounced cross-reactivity-related side effects of the vaccine. Importantly, all samples were collected in the pre-Omicron period but showed noticeable responses to the receptor-binding domain (RBD) of the Omicron spike protein. Thus, using the new express Ab-profiling system, we confirmed the inter-variant cross-reactivity of the anti-SARS-CoV-2 Abs and demonstrated the relative potency of the vaccines against new VOCs.

## 1. Introduction

Humoral immune response profiling is an important part of diagnosing and monitoring coronavirus infectious disease (COVID-19). Microarrays with immobilized antigens have recently emerged as a promising alternative to enzyme-linked immunosorbent assays (ELISA) kits in profiling serological responses to bacterial and viral infections [1,2]. The key advantage of the microarrays is their compatibility with high-throughput multiplex testing. They are also increasingly considered for related purposes, such as biomarker discovery [2].

Microarray systems are typically designed for reflectometry-based detection [3] or fluorescence detection using labelled secondary Abs [4]. They can be implemented in a multiplex version, e.g., for parallel monitoring of immune responses to several related viruses [2]. Notable examples include microarray-based profiling of the Ab responses to SARS-CoV, SARS-CoV-2, MERS-CoV, etc., which have provided evidence for immunological cross-reactivity between endemic viruses [3,5]. The multiplex approach has also given rise to the development of peptide chips, with immobilised overlapping fragments (20–100 a.a.) of antigen proteins, for the identification of key linear epitopes, recognised by neutralising Abs [6]. This technology has been employed successfully to map the epitopes of SARS-CoV-2 structural proteins [5,7,8]. Of the four structural proteins (namely spike—S, nucleocapsid—N, membrane—M, and viral envelope protein—E), only S and N elicited strongly neutralising Ab response [9,10]. Typical ELISA kits and microarrays are designed for the detection of Abs against full-length N, full-length S, or the receptor binding domain (RBD) of S. Most commercially available microarrays (also referred to as chips) only contain the antigens of early SARS-CoV-2 strains and are thus inapplicable for testing the effects of recent virus mutations.

In-house fabrication of protein arrays of interest can be performed based on standard glass slides or other materials compatible with optical detection. They are typically functionalised with epoxy, carboxy or amino silanes, enabling subsequent covalent protein attachment. Alternatively, the chip slides may be rendered hydrophobic (e.g., covered with polyethylene glycol, nitrocellulose or dextran) for non-covalent protein immobilisation. Nitrocellulose is probably the most popular coating for non-covalent protein attachment, due to its high sorption capacity [11]. Limitations of respective array systems include non-specific sorption of Abs and other serum proteins, which can be overcome to some extent by optimizing blocking conditions. Protein immobilisation is usually performed by contact printing [12,13].

Recently, some have described a new facile microarray fabrication technique, which includes covalent protein immobilisation on activated silane-coated glass slides via programmed spotting by a micro dispenser [14]. Here, the proposed technique [14] was applied to produce arrays with minimal and expanded SARS-CoV-2 antigen panels. The microarray system with a minimal antigen panel was validated for SARS-CoV-2 detection by profiling Abs in the sera of COVID-positive and control (COVID-negative) groups of donors. It was also used to compare Ab levels elicited by two vaccines of different types—namely, the replication-deficient adenovirus vector-based vaccine Sputnik V [15,16], and the inactivated virus (whole-virion)-based CoviVac Russia COVID-19 [16]. The expanded panel was used to predict vaccine efficiency against late SARS-CoV-2 variants of interest/concern and test Ab cross-reactivity with human proteins, as well as inter-strain cross-reactivity.

## 2. Results

### 2.1. Operation Principle of the Microarray System and the Design of the Antigen Panel

**Figure 1:**
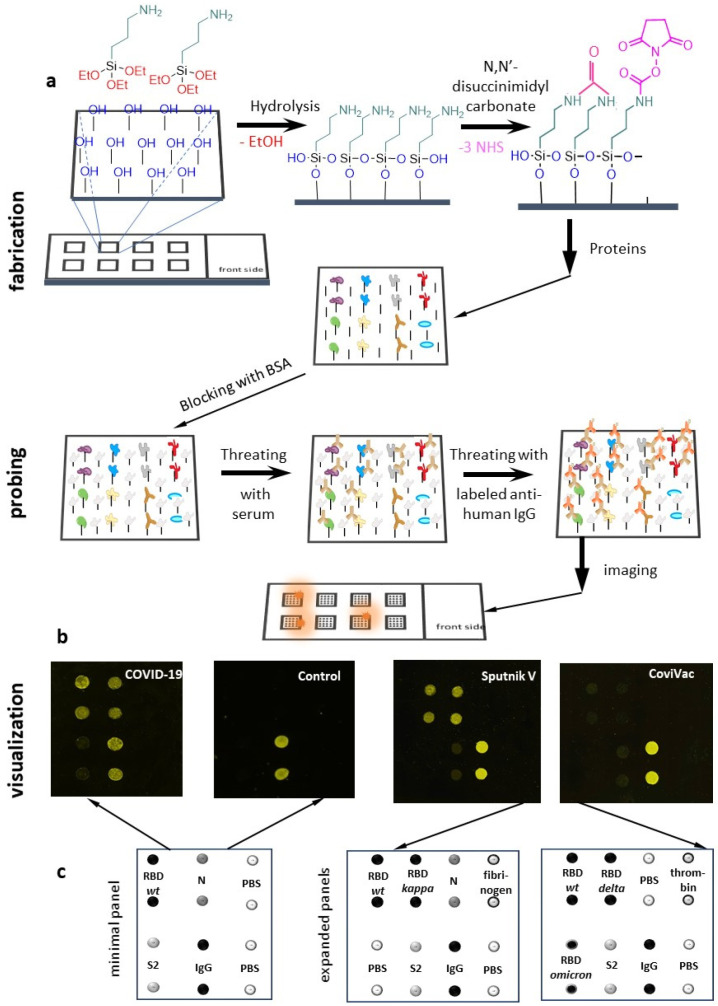
Microarray design and operation principle. (**a**) Schematic representation of the array fabrication scheme and the Ab profiling pipeline; (**b**) Examples of array scans; (**c**) The panels of antigens for Ab profiling.

The working principle of the proposed microarray system (Figure 1) was rather straightforward: recombinant viral antigens of interest and control proteins were immobilised on the glass support, probed with diluted sera, and the Ab-antigen complexes were visualised using fluorescently labelled secondary Abs. Fluorescence intensity in various spots of the microarray slide was quantified and normalised using the intensity in the internal standard spot. For semi-quantitative detection, each antigen was analyzed in two technical and two biological repeats. For quantitative analysis, the number of replicates should be increased to further consolidate the method. Phosphate buffer saline (PBS) was used as a negative control. 

To test whether the microarray system enables accurate discrimination between infected and healthy donors and to justify its consideration for high throughput diagnostics among non-vaccinated patients, we developed a minimal antigen panel. It included three structural viral proteins that reportedly elicit Ab responses—namely, subunit S2 of the spike protein, RBD of the spike protein and the nucleocapsid (N) protein. These three antigens were immobilised on array spots, along with the internal standard (IgG) and the negative control (PBS). Because all samples from COVID+ and COVID- groups were collected in the early- and pre-pandemic era, respectively, wild-type classical (Wuhan) RBD was used in the minimal antigen panel for array validation.

The expanded panel of antigens was developed for subsequent approximate evaluation of the inter-strain cross-reactivity of the Abs to generally predict vaccine potency against new strains. It included RBDs of early (Kappa) and late (Omicron and Delta) SARS-CoV-2 VOI/VOCs. To illustrate the prospects of the microarray system in broader cross-reactivity tests, we also included human blood clotting-related proteins, fibrinogen and thrombin, into the expanded panel of targets. Representative scans of the arrays, with minimal and expanded antigen panels, are shown in Figure S1.

### 2.2. Microarray Validation for Ab Profiling

**Figure 2:**
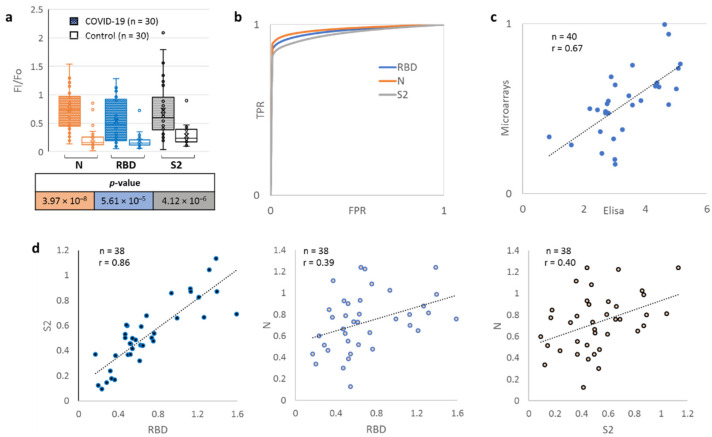
Microarray validation and comparison with ELISA. (**a**) Profiling responses to structural SARS-CoV-2 proteins using protein microarrays; (**b**) ROC curves for N, S2 and RBD proteins; (**c**) Correlation between microarray-based and ELISA-based evaluations of RBD-recognizing Abs and ELISA data; (**d**) Correlation between RBD-, S2- and N-recognizing Abs (microarray-based data).

To validate microarray-based semi-quantitative detection of anti-SARS-CoV-2 Abs, sera samples from two groups of donors (COVID-negative and COVID-positive) (Figure 2a) were profiled, using the minimal antigen panel. The COVID-negative set contained 30 samples from healthy donors collected in the pre-COVID era (2018). The COVID-positive set contained 30 samples from 22 donors with RCR-confirmed SARS infection, minor to moderately severe symptoms and the ELISA-confirmed presence of SARS antigens in sera samples. ELISA data showed no apparent dependence on either the hospital of sample collection, donor age or gender. 

Median normalised Ab levels (F/Fo) in the COVID-positive and COVID-negative groups differed substantially, suggesting the two groups can be reliably distinguished based on this parameter. This result was verified by ROC curve analysis (Figure 2b), which confirmed the microarray-based method to be valid for COVID diagnostics. Method sensitivity, selectivity and accuracy were calculated using the ROC-derived optimal threshold levels of Abs to N, S2 and RBD proteins (Table S2). Consistent with previous reports [17], the highest accuracy (97%) was achieved using the N-based classifier.

To further verify the N/S2/RBD-based classifiers, we analysed an additional sample set, comprising ten PCR-confirmed SARS-positive and ten SARS-negative samples. All of these were classified correctly. Finally, the correlation between microarray-based and ELISA-based evaluations of RBD-recognising Abs was verified (Figure 2c). The Pearson criterion value of 0.67 suggests a reasonably good correlation, considering that both methods are semi-quantitative. Regarding microarray-based data on Abs against different structural proteins, a good correlation was observed between RBD and S2 (the Pearson criterion value was equal to 0.86), and none showed a significant correlation with N (Figure 2d).

### 2.3. Microarray Application for Vaccine Comparison

To characterise the relative strength of Ab responses elicited by the infection and vaccines, we compared distribution and median levels of Abs to N, S2 and RBD (Wuhan variant) detected for the respective groups of donors in the microarrays assays. For CoviVac-treated donors, we could only obtain sera samples collected 1–2 months after vaccination (Ab levels reach a maximum within this period, according to reported ELISA data [18]), whereas, for Sputnik V-treated donors, sample collection was performed in two series: 1–2 months after vaccination (for early response assays) and 3–6 months after vaccination (for late response assays). The late response period limit was selected based on previous reports [19] and the recommended revaccination rate. 

**Figure 3:**
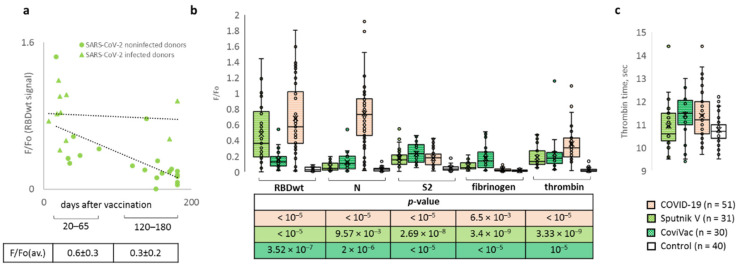
Microarray application for vaccine comparison and the analysis of cross-reactivity with autoantigens. (**a**) Early and late Ab responses of pre-infected and COVID-free donors in the Sputnik V-vaccinated group; (**b**) Responses of convalescent (COVID-19) and vaccinated donors to structural SARS-CoV-2 proteins, human fibrinogen and thrombin; (**c**) Thrombin time values for convalescent, vaccinated and healthy donors.

Although anti-RBD response tended to decrease with increasing time post-vaccination (average Ab levels equal to 0.6 ± 0.3 and 0.3 ± 0.2 for early and late Sputnik V-induced responses, respectively; Figure 3a), this dependence was minor (*p*-value = 0.0012), supporting prolonged vaccine effects in the majority of patients. In addition to vaccination time, we considered possible impacts of other factors on Ab levels, including the donor’s age and gender, and observed no apparent correlations. The only factor that had a major impact on the post-vaccination Ab level was previous infection (Figure 3a). Although all donors had been COVID-free for at least 6 months prior to vaccination, 32% had been diagnosed with COVID-19 at an earlier point (6–12 months prior to vaccination), and these donors demonstrated enhanced Ab response (the average anti-RBD level was equal to 0.8 ± 0.2 for this group versus the average anti-RBD level equal to 0.3 ± 0.2 for the group lacking a pre-vaccination COVID diagnosis).

For CoviVac-treated donors, no information on previous diagnoses was available. Thus, one cannot exclude a preliminary infection-related bias in subsequent data analysis. However, because the samples for CoviVac and Sputnik V groups were collected almost in parallel (May–June and August–September 2021, respectively), the ratio of pre-infected and COVID-free donors can be assumed to be more or less similar in these groups. Thus, the comparison of responses to Sputnik V and CoviVac was performed irrespective of the pre-vaccination diagnoses (Figure 3b).

Sputnik V induced higher levels of anti-RBD Abs than CoviVac (*p*-value = 0.000028). Both groups were significantly superior to the COVID-negative group (Figure 3b) but inferior to the COVID-positive group (*p*-value = 0.000003). Collectively, data on the anti-RBD responses in Sputnik V and COVID-positive donor groups agree with the previously reported trend: vaccination < infection < infection with subsequent recovery and vaccination [20]. As expected, levels of Abs to other structural proteins in the CoviVac group (whole virion-based vaccine) were higher than those in the Sputnik V group (RBD-encoding DNA vaccine). Because anti-N Abs and some anti-S Abs cannot neutralise the virus and may cause undesired effects, especially in the case of cross-reactivity with human proteins, cross-reactivity studies were performed.

### 2.4. Microarray Application for Cross-Reactivity Analysis

With a high degree of probability, it can be argued that the S and N proteins are involved in the mimicry of human epitopes, i.e., they may provoke an autoimmune response [21,22,23]. Because blood-clotting aberrations, especially thrombosis, are among the most commonly reported vaccine side effects [24,25,26], and the underlying mechanisms imply autoimmunity, we selected two key proteins of the blood clotting system, namely thrombin and fibrinogen, as control human antigens for cross-reactivity tests. Alignment of the amino acid sequences of the SARS-CoV-2 S and N proteins with human fibrinogen and (pro)thrombin revealed multiple 9–14 a.a. fragments of substantial (≥30%) similarity (Figures S2–S9, calculated in VectorBuilder Sequence Alignment), supporting the mimicry hypothesis.

Cross-reactivity with fibrinogen was pronounced only in the case of CoviVac-induced immunity (Figure 3b), so we assumed some epitopes present in CoviVac, but not Sputnik, to be responsible for this effect. These epitopes may have arisen in the process of virion inactivation, because infection with the wild-type virus (COVID-positive group) induced no fibrinogen-recognising Abs. In the case of thrombin, a small autoimmune response was observed in all donor groups, except for the control (COVID-negative) one. It was questioned whether this small autoimmune response caused any coagulation abnormalities, and the thrombin time of fibrinogen-enriched sera was measured in samples using standard in vitro tests (Figure 3c). Thrombin time is the time it takes for thrombin to generate fibrin clot by cleaving fibrinogen. This parameter was only slightly increased in COVID-19 and CoviVac groups, suggesting that virus/vaccine-induced levels of anti-thrombin Abs are insufficient to cause noticeable clotting aberrations.

**Figure 4:**
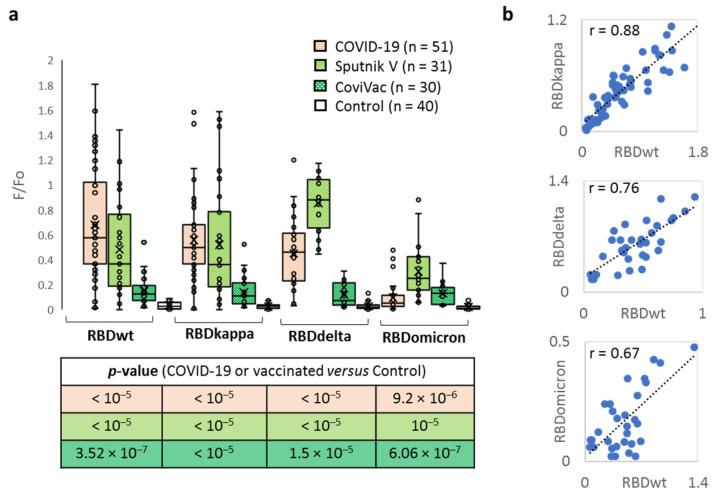
Microarray application for the analysis of inter-strain cross-reactivity. (**a**) Responses of convalescent (COVID-19) and vaccinated donors to RBDs of different SARS-CoV-2 variants; (**b**) Correlation between the levels of Abs against RBD of different SARS-CoV-2 variants.

Finally, to predict the potency of Sputnik and CoviVac, both of which had been initially developed to combat the Wuhan SARS-CoV-2 variant, against new strains, we tested all donors’ responses to the recently emerged VOI/VOC (Figure 4a). RBDs of the Kappa (B.1.617.1), Delta (B.1.617.2) and Omicron (B.1.1.529) SARS-CoV-2 variants correlated with the Wuhan variant, with Pearson coefficients of 0.88, 0.76 and 0.67, respectively (Figure 4b), supporting substantial inter-strain cross-reactivity. Average anti-Kappa RBD Ab levels decreased in the following order: COVID-positive group > Sputnik > CoviVac, showing the same trend as anti-Wuhan RBD Abs, and all of them exceed the Ab level in the control (COVID-negative) group. Abs against RBDs of Delta and Omicron showed a different trend, and the top response (particularly pronounced for Delta) was observed in the Sputnik group. With respect to the immune evasion potential of new VOI/VOC, key results can be summarised as follows:

- Kappa RBD showed zero to minor evasion of anti-Wuhan variant Abs in both convalescent and vaccinated donors;
- Delta showed minor Ab evasion in convalescent and CoviVac-vaccinated donors, but was efficiently recognised by the Abs of Sputnik V-vaccinated donors;
- Omicron evaded all Abs to a high extent, except that its signal was similar to the Wuhan variant in the CoviVac group.

It can be concluded that, despite the substantial propensity of the virus for immune evasion, both tested vaccines may provide reasonably good protection against new VOC/VOI. 

## 3. Discussion

The proposed microarray assays made it possible to distinguish SARS-CoV-2-positive donors 2–33 days after the onset of moderately severe symptoms with an accuracy of up to 97% (Figure 2, Table S2). They proved comparable to known multiplex immunoassays [27,28,29,30,31] in terms of both sensitivity (84, 89 and 90% for S2, RB, and N, respectively) and specificity (98, 95 and 98% for S2, RBD and N, respectively). They also appear slightly superior to the recently reported hydrogel-based microarrays [32], which showed 85–95% accuracy in analogous tests. It should be noted, however, that the disease onset time was unknown in the reported hydrogel microarray assay, so comparison with our new microarray system might be biased. Regardless, available data support the validity of the new microarray-based assays for serological tests.

Importantly, the new microarrays and the previously reported alternatives were calibrated using the samples from the onset of the pandemic, which was supposedly characterised by moderate virus heterogeneity. Reduced sensitivity of the Abs detected in respective sera samples to the later strains (Figure 3) can thus indicate SARS-CoV-2 immune evasion and an increased probability of reinfection. Our data imply moderate anti-RBD Ab evasion of B.1.617 (Kappa and Delta) and high Ab evasion of B.1.1529 (Omicron). These findings agree with previous reports on convalescent donors [33] and multiple monoclonal Abs, including those currently in clinical use and trials. For instance, therapeutic neutralising Abs Bamlanivimab, Etesevimab, Casirivimab, Imdevimab, Tixagevimab and Regdanvimab lost neutralising activity against Omicron [34], and Bamlanivimab also lost the activity against Delta [34,35,36]. Sotrovimab is the only commercially available Ab that proved rather potent against both strains [34]. 

Unlike Abs from convalescent donors, vaccine-elicited Abs showed no clear preference for Wuhan RBD (Figure 4b). The only trustworthy difference was observed between responses to Delta and Omicron in the Sputnik V group (the Delta: Omicron response ratio was similar to that in convalescent donors), while the CoviVac group showed similarly modest responses to all SARS-CoV-2 variants. Surprisingly enough, the Sputnik V-induced Ab response to Delta seemed stronger than that to Wuhan. This might be due to pre-vaccination infections, which cannot be easily tracked accurately but facilitate prolonged maintenance of high Ab levels (Figure 4a). According to the literature [20], vaccination with Sputnik V induced higher Ab levels in patients that had recovered from COVID-19 than in non-infected patients, and both variants were superior to infection without vaccination. Indeed, anti-RBD responses in the Sputnik V group were higher than those in the COVID-19 group in most cases (Figure 4b). The rather modest potency of CoviVac compared to Sputnik V is also in line with previous reports [16,37,38]. Other inactivated virus-based vaccines (CoviVac analogues), such as BBIBP-CorV or Covaxin [39,40], are also reasonably potent against new strains, but appear generally inferior to “genetic” vaccines, including mRNA-based vaccines, such as the Pfizer-BioNTech COVID-19 vaccine, and adenoviral vector vaccines (Sputnik V analogues), such as ChAdOx1-S/nCoV-19 [37,38]. 

Along with potency against classical and emerging VOCs, one should take into account the possible cross-reactivity of vaccine-elicited Abs with autoantigens. Although both “genetic” and inactivated virus-based vaccines rely on anti-RBD Abs, the latter may provide a broader spectrum of autoantigen-resembling viral epitopes (e.g., N-derived ones). However important, cross-reactivity with autoantigens is unlikely the only cause of common vaccination side effects. Reported data [41] show that adenoviral DNA can undergo alternative splicing, which may lead to the synthesis of soluble spike protein variants. Soluble spike proteins, as well as the highly specific blood flow conditions in the central venous sinus of the brain, supposedly account for rare but severe events after vaccination, as observed with ADZ1222/Vaxzevria and Johnson & Johnson vaccines [42,43].

In this study, in some cases, vaccinated donors showed a small autoimmune response to fibrinogen; in those vaccinated with CoviVac, the level of Abs was slightly higher than in those vaccinated with Sputnik V. As for Abs to thrombin, they were observed both in those who recovered from COVID-19 and vaccinated with Sputnik V and CoviVac, within acceptable limits. 

The data obtained by Russian scientists [44] made it possible to assert that indicators of plasma hemostasis, such as the stationary and initial thrombus growth rate, growth retardation, density and size of the clot, and the time of appearance of spontaneous thrombi, do not undergo statistically significant changes within 42 days following the introduction of the first component of vaccines Sputnik V and CoviVac. Since these indicators reflect the state of plasma hemostasis, it can be concluded that, during the observation period, no laboratory signs of significant changes in hemostasis were found in those vaccinated with the Sputnik V and CoviVac vaccines, compared with the indicators at the initial observation point.

## 4. Materials and Methods

### 4.1. Sera Samples

The donors enrolled in this study were separated into four groups:

- 40 donors who had not encountered SARS-CoV-2 (their sera samples were collected in 2018);
- 31 donors who underwent vaccination with the replication-deficient adenovirus vector-base vaccine Sputnik V 20–65 days or 120–180 days prior to sera sample collection;
- 30 donors who underwent vaccination with the whole virion-based vaccine CoviVac 35–65 days before sera sample collection;
- 22 convalescent patients were diagnosed with COVID-19 2–33 days prior to sera sample collection (Table S1).

In the case of the convalescent donors, sera samples were collected at different stages of the disease progression: at the time of admission to the hospital, 48 h after hospitalisation, 7 days after hospitalisation and at the time of discharge from the hospital. 

All human donors voluntarily gave informed consent prior to being enrolled in the study and provided their consent for study publication.

To separate serum from blood cells in the patient blood sample, a vacuum tube (Clot Activator Tube) was left at room temperature for 1 h. The clot was separated from the walls of the test tube by a circular motion of the capillary of a sterile Pasteur pipette. A vacuum tube was centrifuged (centrifugation conditions: 1300× *g* for 10 min). The serum was carefully pipetted into a sterile tube and frozen (−20 °C).

### 4.2. Recombinant Proteins

Recombinant SARS-CoV-2 nucleocapsid protein (N) and the S2 subunit of the spike protein were produced in *E. coli* transformed with the pET-22b-based antigen-encoding plasmids, and RBD was produced in Expi293F cells (Thermo Fisher Scientific, Waltham, MA, USA) transformed with the pcDNA-3.4-based plasmid (Thermo Fisher Scientific, Waltham, MA, USA). To obtain the antigen-encoding plasmids, viral RNA was isolated from the scraping samples of SARS-positive patients (Wuhan variant). The RevertAid RT Reverse Transcription Kit (Thermo Fisher Scientific, Waltham, MA, USA) was used to carry out the reverse transcription reaction. DNA fragments encoding viral antigens were obtained by PCR amplification of viral cDNA, incorporated into the modified (signal peptide-lacking) pET-22b plasmid or pcDNA-3.4. plasmid, and cloned in *E. coli*. Isolated RBD/S2/N-His-tag-coding plasmid structures were confirmed by Sanger sequencing.

The *E. coli* BL21 (DE3) strain was transformed by N and S2-coding plasmids, and the Expi293F cell line was transfected by the RBD-coding plasmid. N protein was accumulated in *E. coli* in a water-soluble form, while S2 was accumulated in the inclusion bodies. RBDs were accumulated in a water-soluble form and isolated from the Expi293F culture liquid. Recombinant C-His6-tagged proteins were purified by metal chelate affinity chromatography. 

To obtain selected RBD variants of interest (VOI) and concern (VOC), the following mutations were introduced into the consensus sequence using site-directed mutagenesis: L452R and E484Q, B.1.617.1 (Kappa) [45]; L452R and T478K, B.1.617.2 (Delta) [46,47,48]; G339D, S371L, S373P, S375F, K417N, N440K, G446S, S477N, T478K, E484A, Q493K, G496S, Q498R, N501Y and Y505H, B.1.1529 (Omicron) [46,48].

### 4.3. ELISA

ELISA was performed as described previously [14] using the SARS-CoV-2 IgG kit (Lytech Co., Ltd., Moscow, Russia), according to the manufacturer’s instructions. In brief, sera samples diluted 1:10 with PBS were placed into a microtiter plate pre-coated with RBD antigens. Following a brief incubation, the plate was washed five times to remove unbound Abs. Then, the solution of horseradish peroxidase conjugated with anti-human IgG was added, and the plates were incubated at 37 °C for 30 min. Finally, 3,3′,5,5′-tetramethylbenzidine (TMB) reagent was added, and the mixture was incubated for an additional 10 min in the dark. The reaction was stopped by acid. Immediately after that, the absorbance of the tetramethylbenzidine charge transfer complex and the diamine terminal oxidation product was measured at 650 nm and 450 nm, respectively. All experiments were performed in duplicate. For each sample, the coefficient of positivity was calculated: CP = ODsample/ODcritical, where ODcritical = ODmean K- + 0.12, and ODmean K- is the average OD for two negative control wells. The sample was marked as COVID-positive for CP > 1.1 and negative for CP < 0.9; CP within 0.9–1.1 was considered equivocal.

### 4.4. Microarray Assays

Microarray fabrication, protein immobilisation, sera probing and Ab-protein complex visualisation were performed according to a recently reported protocol developed by our group [14]. Briefly, chemically polished Pyrex glass slides were modified with aminopropyltriethoxysilane and then functionalised for subsequent protein immobilisation via primary amino groups by using a bifunctional linker (N,N′-disuccinimidyl carbonate). Immobilisation of the proteins was performed by spotting their 0.1 mg/mL solutions in PBS (pH 7.4) on the slide surface using an iTWO-300P piezo-driven micro-dispenser (M2-Automation Systems, Berlin, Germany). The free slide surface was blocked with a 1 mg/mL BSA solution in PBS. Sera samples were slowly thawed, diluted 1:10 with a Tris-HCl buffer (pH 7.5), were applied onto the slide surface and incubated for 30 min at room temperature. After that, the slides were rinsed with the same buffer, treated with a solution of Alexa Fluor 546-labeled secondary Ab, incubated for 1 h in darkness at room temperature, rinsed again and scanned using a Nikon Eclipse Ti2 microscope (Nikon, Tokyo, Japan) equipped with a DS-Qi2 highspeed camera. Key modifications of the reported assay procedure concerned the design of the antigen panel and data processing (signal normalisation). In addition to the main structural proteins of the initial (Wuhan) SARS-CoV-2 variant and the negative control (PBS), the new panel included IgG (internal standard), recent VOI/VOC RBDs and antigens of the human blood clotting system. The fluorescence signal in each spot (F) was calculated as an average fluorescence intensity minus the average background. For each protein, two replicates were averaged, and a normalised signal was calculated as F/Fo ± SD, where Fo was the average IgG signal.

### 4.5. Coagulation Assays

To obtain fibrinogen-enriched sera for thrombin time tests [49], commercially available purified human fibrinogen (Sigma-Aldrich, St Louis, MO, USA) was dissolved in 0.9% saline to a final concentration of 20 mg/mL and mixed 1:1 with the donor’s sera. Coagulation tests were performed using an APG4-03-Ph haematology analyser (EMCO, Moscow, Russia) and thrombin reagent for thrombin time determination (Renam, Moscow, Russia), following the standard protocol. Each fibrinogen-enriched sera sample was incubated at 37 °C for 2 min with stirring. An equal volume of a thrombin reagent solution (6 IU/mL) was then added at room temperature, and the thrombin time was measured. All experiments were performed in triplicate.

### 4.6. Statistics

The significance of the difference between Ab responses in various groups of donors was verified using z-statistics. Relative Ab levels quantified as normalised fluorescence signal values (F/Fo) in the microarray assays showed non-normal distributions in most donor groups. The statistical significance of the difference between the groups was therefore verified using the Mann-Whitney U-test. All normalised signal values in the control group and the group of interest were ranked and compared pairwise to determine U^group of interest^ and U^control group^. These scores indicate the total number of times F/Fo^group of interest^ exceeds F/Fo^control group^ and vice versa. The smaller of the two scores (U) was used to calculate the z-score.

(1) $$z=\left(U-\frac{n^{\mathrm{group}\;of\;interest}\;\cdot \;n^{\mathrm{control}\;\mathrm{group}}}{2}\right)/\sqrt{\frac{n^{\mathrm{group}\;of\;interest}\;\cdot \;n^{\mathrm{control}\;\mathrm{group}}\left(N+1\right)}{12}}$$

Normal approximation, with a respective z-score equation, was employed because the total sample size (*N* = *n^group of interest^* + *n^control group^*) was large enough (>20) in all cases.

To evaluate the performance of microarray-based diagnostic tests, receiver operating curves (ROC) were obtained for N/S2/RBD-related F/Fo values as infection markers. In each case, experimental data were fitted to an exponential model

TPR = R·FPR^1/E^ + (1 − R)·[1 − (1 − FPR)^E^] (2)

where TPR is the true positive rate, FPR is the false positive rate, the parameter E indicates the curve “height” along the negative diagonal and the parameter R indicates the “skewness” of the curve with respect to the negative diagonal. The optimal F/Fo threshold was selected graphically and used to calculate classification sensitivity, selectivity and accuracy.

Pairwise covariances of Ab responses to different viral proteins or S protein RBDs of different VOI/VOC, evaluated by microarray assays and covariances of ELISA and microarray data, were characterised using Pearson correlation coefficients.

## 5. Conclusions

This work has demonstrated the applicability of new protein microarray assays for measuring the levels of anti-SARS-CoV-2 IgG Abs in human blood sera. Statistical analysis confirmed their excellent analytical performance in terms of precision, sensitivity and specificity. Microarray assays revealed a significant difference between anti-VOC Ab levels in recovered patients, Sputnik V-vaccinated donors and CoviVac-vaccinated donors. The assays also confirmed inter-strain cross-reactivity of the Abs, and revealed minor cross-reactivity with blood clotting regulators. The results support high and moderate efficacy of Sputnik V and CoviVac vaccines, respectively, and suggest that vaccination with Sputnik V might provide stronger protection from recent SARS-CoV-2 variants than natural immune response to early variants.

## Supplementary Materials

The following supporting information can be downloaded at: https://www.mdpi.com/article/10.3390/ijms232113220/s1.
