# Supplementary material for: Microarray Profiling of Vaccination-Induced Antibody Responses to SARS-CoV-2 Variants of Interest and Concern
Source: Int J Mol Sci. 2022 Oct 30;23(21):13220. doi: 10.3390/ijms232113220 (PMC9655829; doi:10.3390/ijms232113220)

Table S1. Detailed information on serum samples tested in this study.

**Patient group**		**n=22**
Gender	Male	12
	Female	10
Median age (years)		59.0
Severity	Mild cases	14
	Moderate cases	6
	Hard cases	2
**Sputnik V group**		**n=31**
Gender	Male	16
	Female	15
Median age (years)		38.4
**CoviVac group**		**n=30**
Gender	Male	5
	Female	25
Age (years)		
**Control group**		**n=31**
Sample collection data (year)		2018

Figure S1. Examples of the array scans

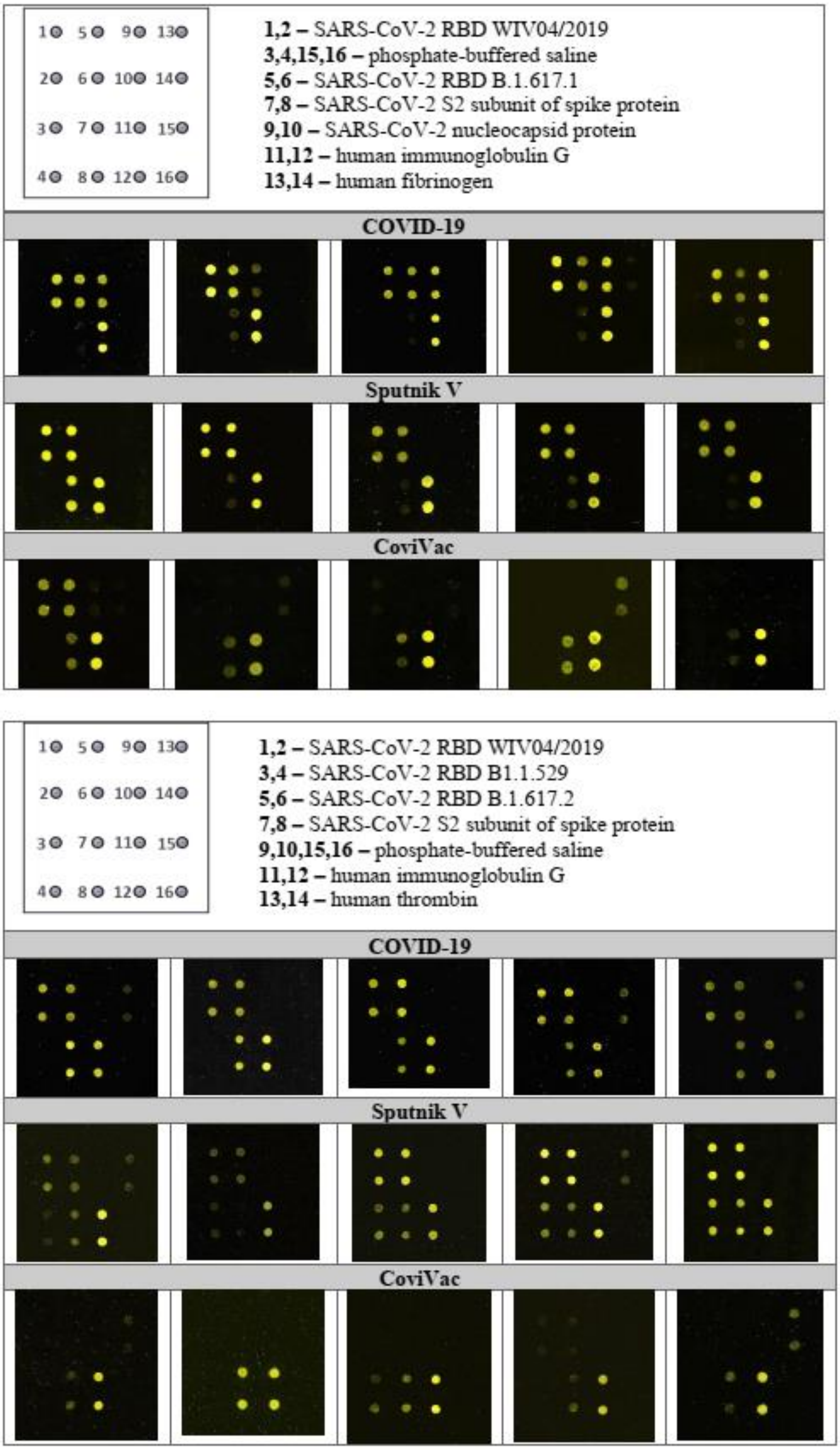

Table S2. Sensitivity, selectivity and accuracy for microarray method

	RBD	S2	N
**COVID-19 (n=60)**			
**True Positives**	29	29	30
**False Positives**	1	1	2
**True Negatives**	26	27	25
**False Negatives**	4	3	3
**Sensitivity**	0.89	0.84	0.90
**Specificity**	0.95	0.98	0.98
**AUC**	0.96	0.93	0.97

Figure S2. The sequence alignment of the SARS-Cov-2 S protein and human fibrinogen (Chain A)

I – Identity; S - Similarity

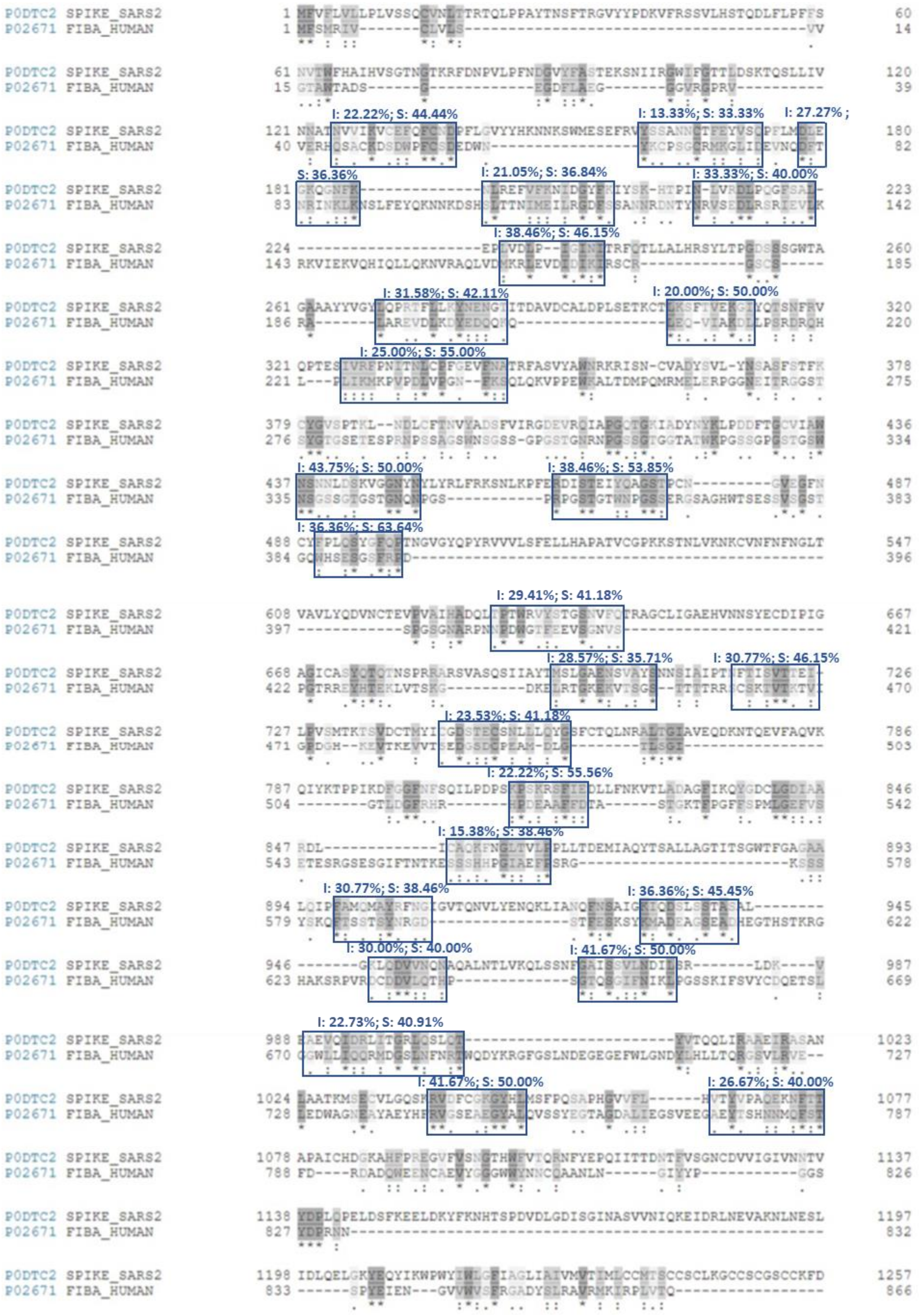

Figure S3. The sequence alignment of the SARS-Cov-2 S protein and human fibrinogen (Chain B)

I – Identity; S - Similarity

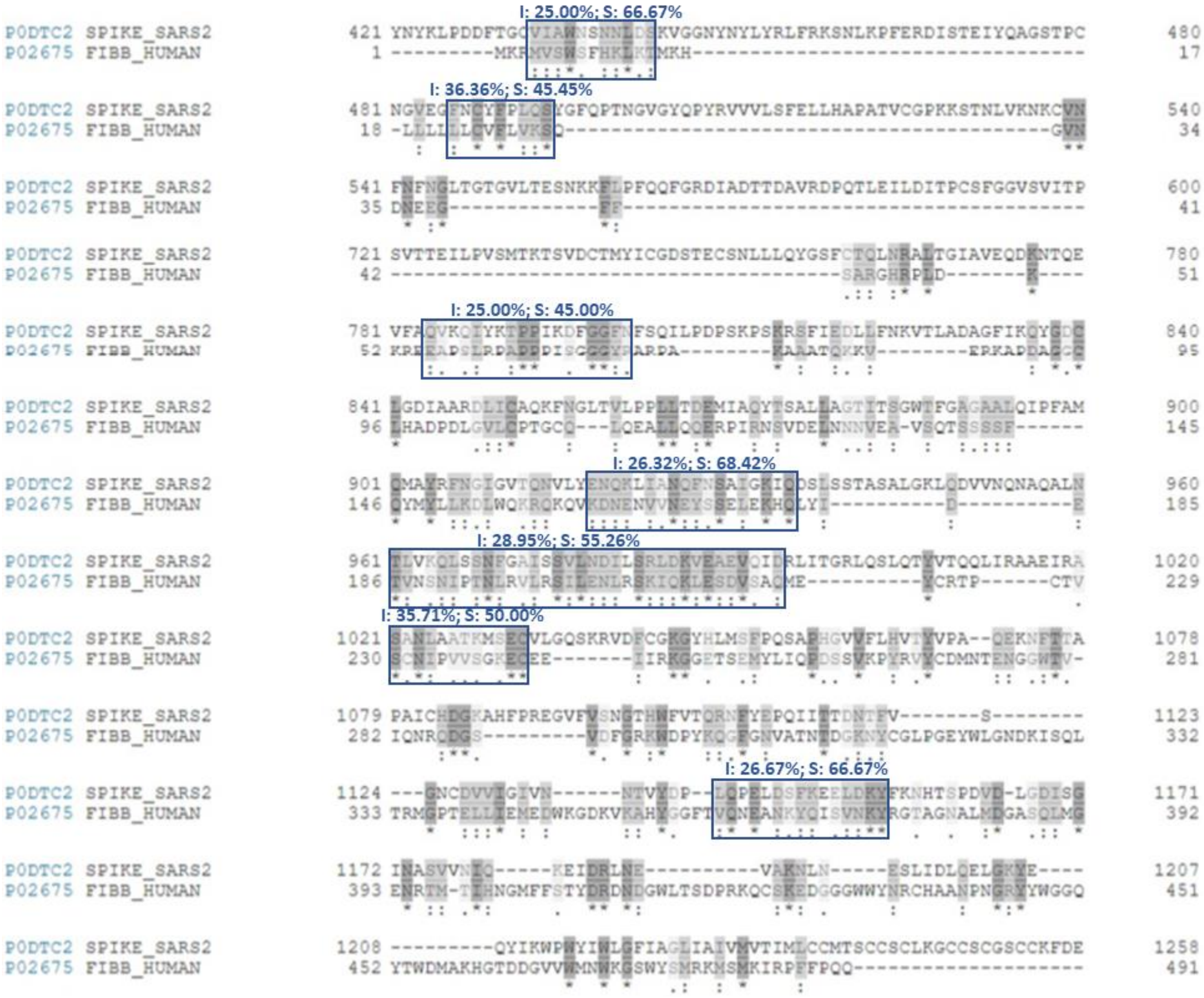

Figure S4. The sequence alignment of the SARS-Cov-2 S protein and human fibrinogen (Chain G)

I – Identity; S - Similarity

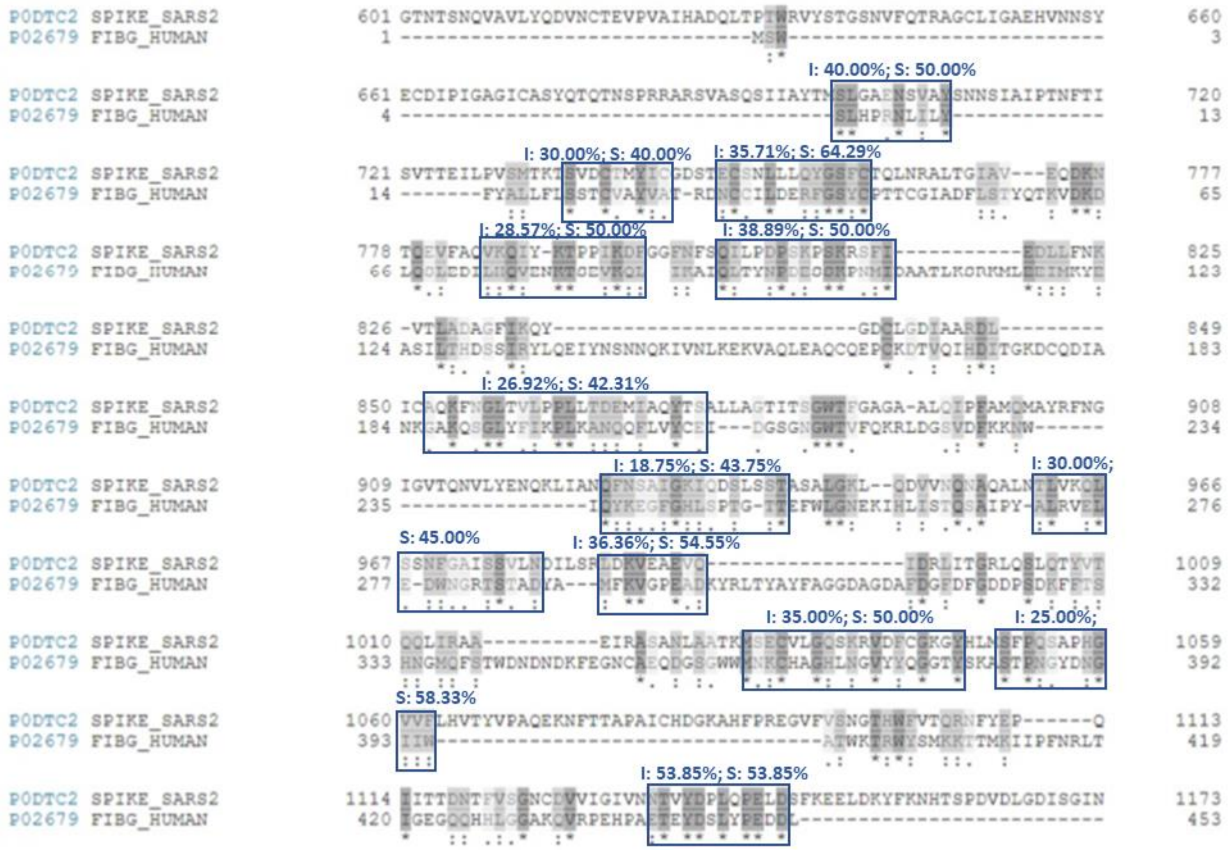

Figure S5. The sequence alignment of the SARS-Cov-2 N protein and human fibrinogen (Chain A)

I – Identity; S - Similarity

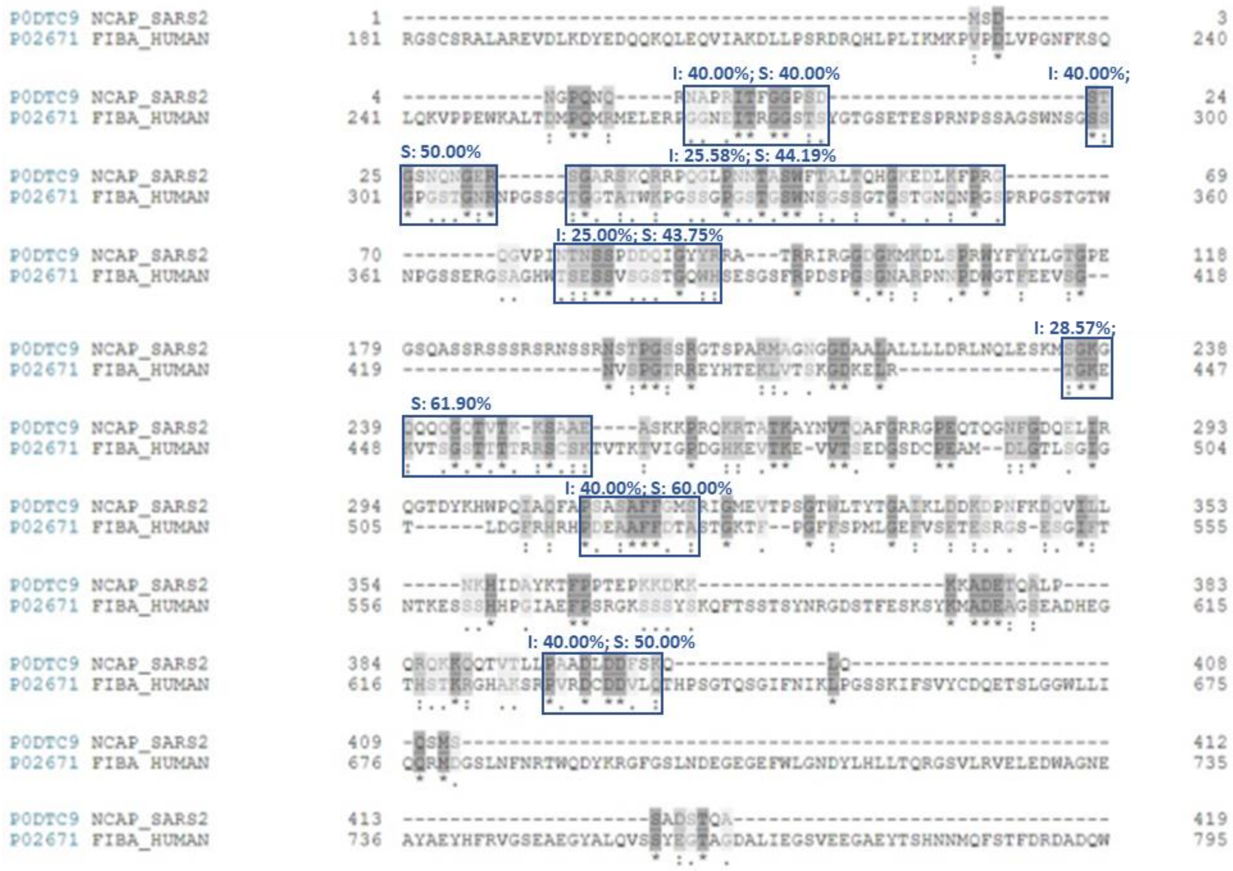

Figure S6. The sequence alignment of the SARS-Cov-2 N protein and human fibrinogen (Chain B)

I – Identity; S - Similarity

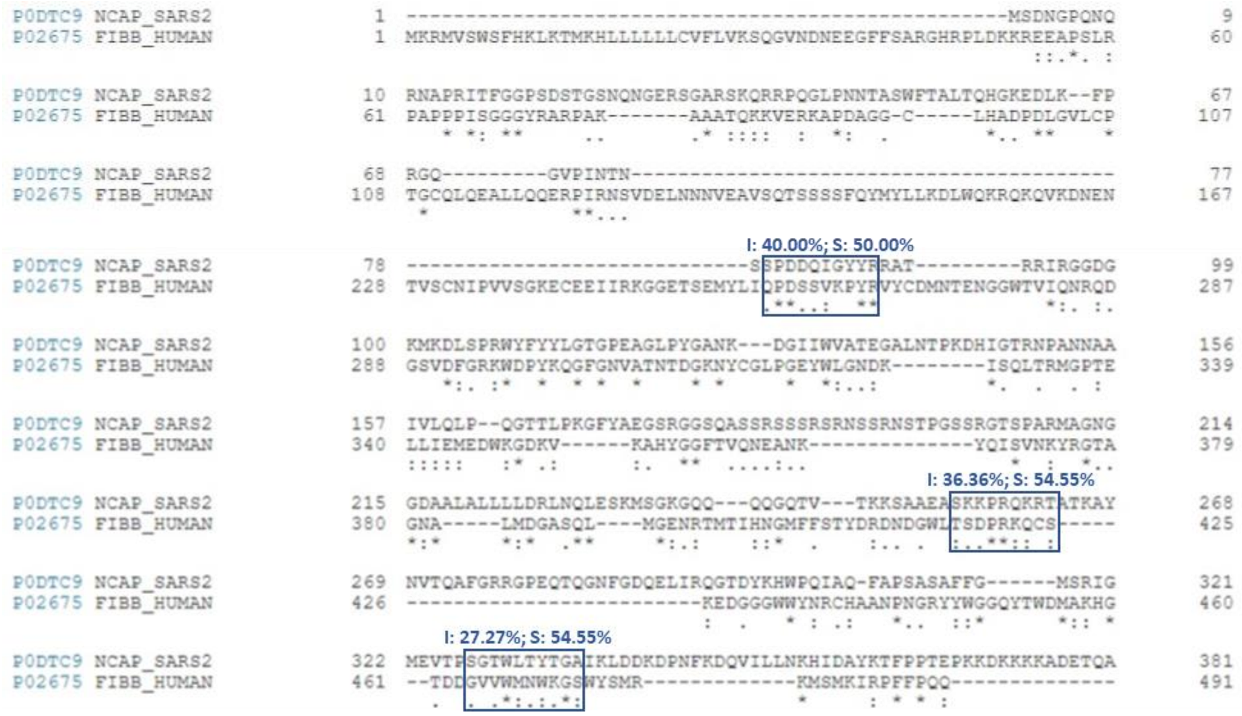

Figure S7. The sequence alignment of the SARS-Cov-2 N protein and human fibrinogen (Chain G)

I – Identity; S - Similarity

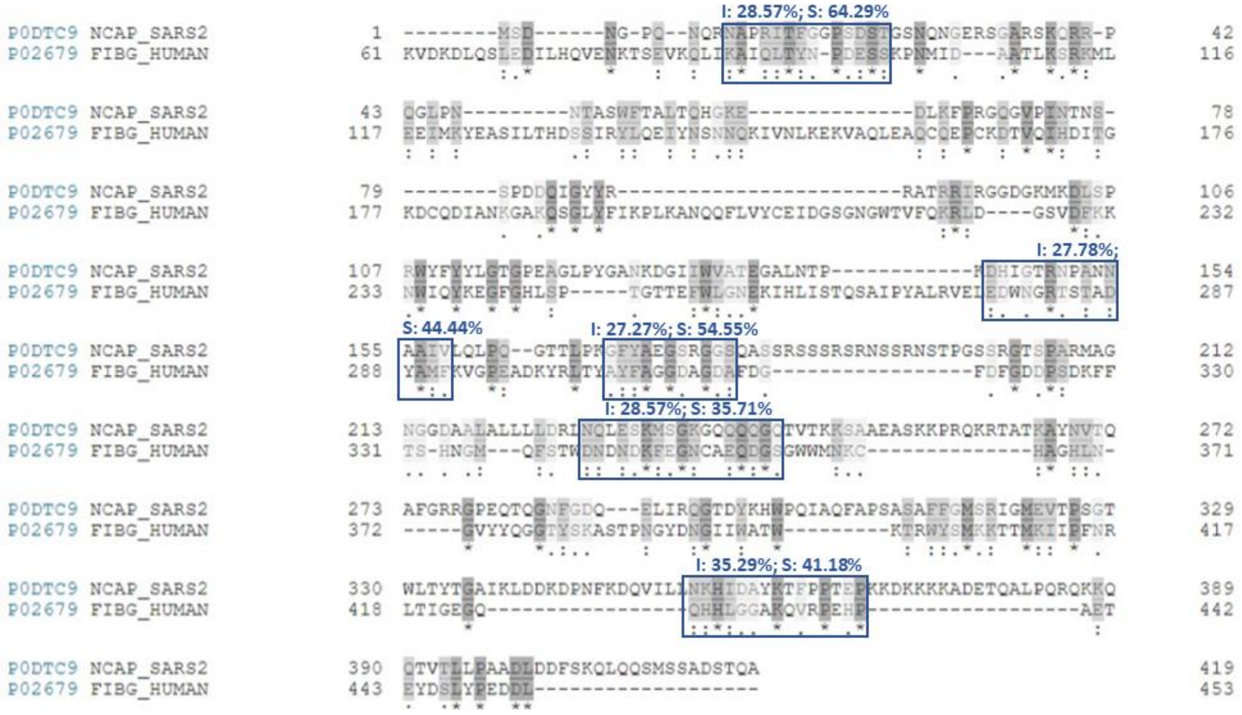

Figure S8. The sequence alignment of the SARS-Cov-2 S protein and human prothrombin

I – Identity; S - Similarity

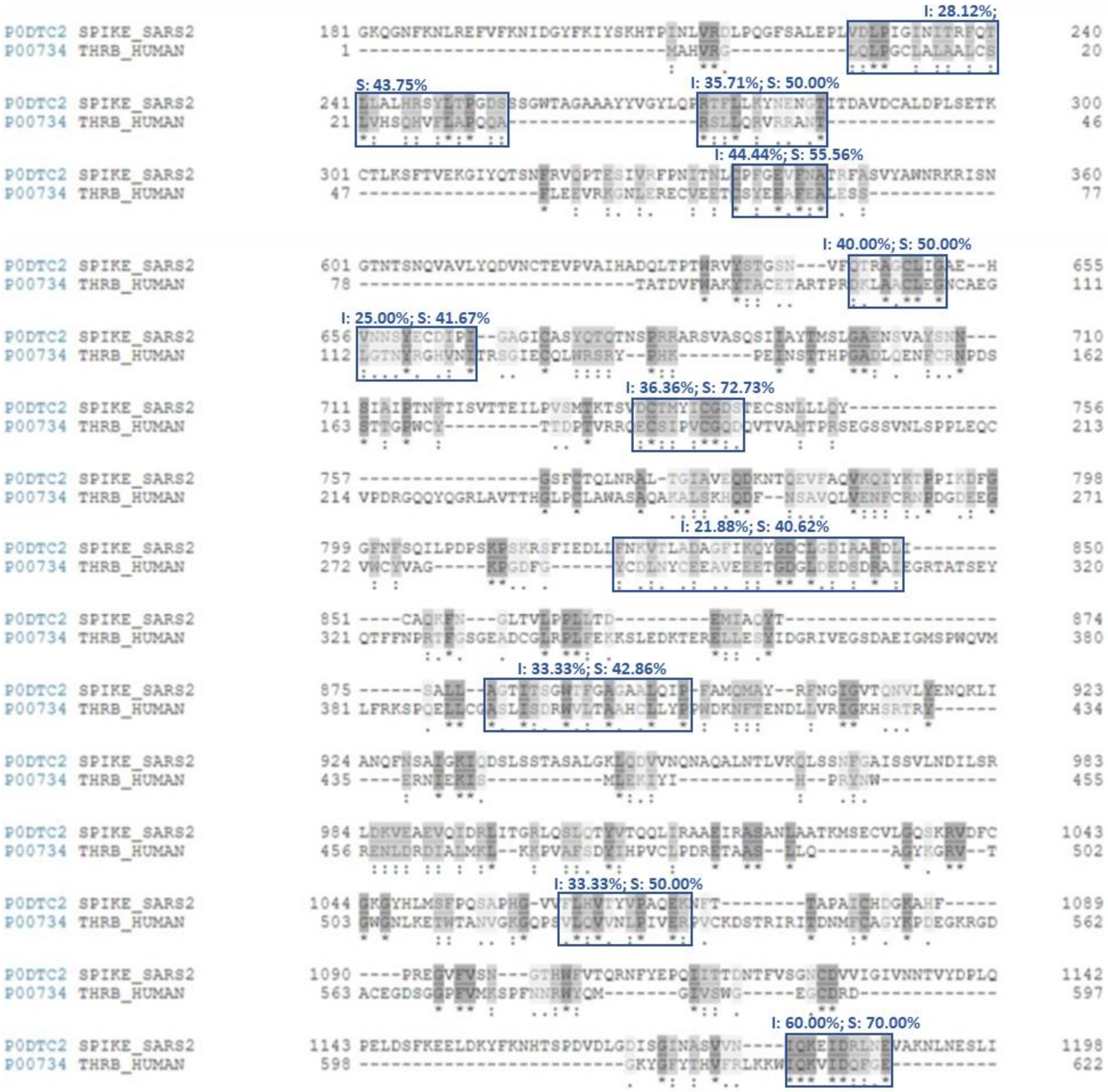

Figure S9. The sequence alignment of the SARS-Cov-2 N protein and human prothrombin

I – Identity; S - Similarity

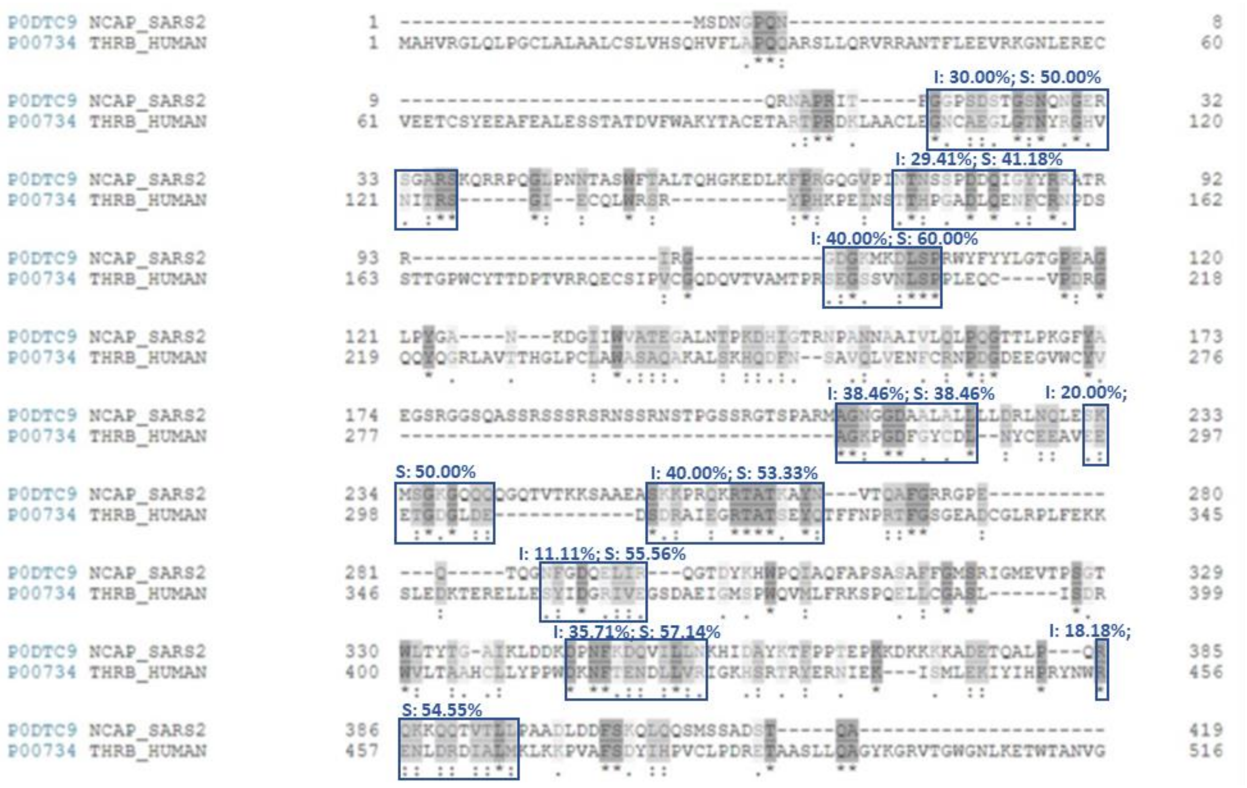

Supplement: Supplementary file 1 [file ijms-23-13220-s001.zip › ijms-1914341-supplementary.pdf]
